# Pharmacokinetics, hemodynamic and metabolic effects of epinephrine to prevent post-operative low cardiac output syndrome in children

**DOI:** 10.1186/cc13707

**Published:** 2014-01-24

**Authors:** Mehdi Oualha, Saïk Urien, Odile Spreux-Varoquaux, Alice Bordessoule, Irène D’Agostino, Philippe Pouard, Jean-Marc Tréluyer

**Affiliations:** 1 Réanimation Pédiatrique. Hôpital Necker Enfants-Malades, Assistance Publique-Hôpitaux de Paris, Université Paris Descartes, Paris, France; 2 CIC-0109 Cochin-Necker Inserm, Unité de Recherche Clinique, Paris Centre Descartes Necker Cochin, Service de Pharmacologie Hôpital Cochin, Assistance Publique- Hôpitaux de Paris et E.A. 3620, Université Paris Descartes, Paris, France; 3 Pharmacologie, Centre Hospitalier de Versailles, Université de Versailles Saint- Quentin-en-Yvelines, Le Chesnay, France; 4 Réanimation Chirurgicale Cardiaque Pédiatrique. Hôpital Necker Enfants-Malades, Assistance Publique- Hôpitaux de Paris, Université Paris Descartes, Paris, France

## Abstract

**Introduction:**

The response to exogenous epinephrine (Ep) is difficult to predict given the multitude of factors involved such as broad pharmacokinetic and pharmacodynamic between-subject variabilities, which may be more pronounced in children. We investigated the pharmacokinetics and pharmacodynamics of Ep, co-administered with milrinone, in children who underwent open heart surgical repair for congenital defects following cardiopulmonary bypass, including associated variability factors.

**Methods:**

Thirty-nine children with a high risk of low cardiac output syndrome were prospectively enrolled. Ep pharmacokinetics, hemodynamic and metabolic effects were analyzed using the non-linear mixed effects modeling software MONOLIX. According to the final model, an Ep dosing simulation was suggested.

**Results:**

Ep dosing infusions ranged from 0.01 to 0.23 μg.kg^-1^.min^-1^ in children whose weight ranged from 2.5 to 58 kg. A one-compartment open model with linear elimination adequately described the Ep concentration-time courses. Bodyweight (BW) was the main covariate influencing clearance (CL) and endogenous Ep production rate (q0) via an allometric relationship: CL(BWi) = θ_CL_ x (BWi)^3/4^ and q0(BWi) = θ_q0_ x (BWi )^3/4^. The increase in heart rate (HR) and mean arterial pressure (MAP) as a function of Ep concentration were well described using an Emax model. The effect of age was significant on HR and MAP basal level parameters. Assuming that Ep stimulated the production rate of plasma glucose, the increases in plasma glucose and lactate levels were well described by turnover models without any significant effect of age, BW or exogenous glucose supply.

**Conclusions:**

According to this population analysis, the developmental effects of BW and age explained a part of the pharmacokinetic and pharmacodynamics between-subject variabilities of Ep administration in critically ill children. This approach ultimately leads to a valuable Ep dosing simulation which should help clinicians to determine an appropriate *a priori* dosing regimen.

## Introduction

Inotropic agents are commonly administered to prevent postoperative low cardiac output syndrome (LCOS) following cardiopulmonary bypass (CPB) in children undergoing open heart surgical repair [1]. According to the PRIMACORP study, milrinone is the first-choice drug [2]. However as described in the European survey EuLoCOS-Paed, preventive drug therapy is highly variable. For instance, epinephrine (Ep), which is cheaper than other commonly used catecholamines, is also used, although evidenced-based data are currently lacking [3,4].

The amplitude of the hemodynamic response to Ep is difficult to predict given the multitude of factors involved and clinical experience suggests broad between-subject variability. This hemodynamic response is primarily dependent on Ep concentrations. However Ep pharmacokinetics has been poorly evaluated in children. Fisher *et al*. suggested linear pharmacokinetics with a lower clearance than that reported in healthy adults, although only six children were included in their study and neither inter-patient variability nor pharmacodynamic effects were described [5]. A recent adult study using population pharmacokinetic modeling highlighted the influence of bodyweight (BW) and disease severity on Ep clearance confirming this variability [6]. These between-subject disparities may be even more pronounced in children.

Pediatric dosages of Ep are usually extrapolated from adult studies. However, postnatal development of cardiac contractility is associated with major changes in the modulatory effect of β-adrenoreceptor signaling. Moreover, differential maturation of the transduction pathways within the cardiomyocyte contributes to age-dependent changes in cardiac responsiveness and sensitivity to agonists [7]. Although much is known regarding the adult physiological and pharmacological effects of Ep, there are very few pediatric studies on Ep pharmacodynamics. Effects of Ep infusion in children have only been described in the neonate, mostly in low birth-weight infants, where effects on heart rate (HR), mean arterial pressure (MAP), plasma glucose and lactate levels were observed [8].

The purpose of the present study was to investigate, using a population approach, the pharmacokinetics and pharmacodynamics of Ep including hemodynamic (HR, MAP) and metabolic effects (plasma glucose and lactate levels) in critically ill children undergoing surgical repair for congenital heart defect, following CPB, as well as associated variability factors [9]. The effects of developmental and other factors on Ep pharmacokinetics and pharmacodynamics were investigated in order to better explain the observed between-subject variabilities and to ultimately suggest individualized dosage regimens.

## Materials and methods

### Setting

This prospective study was conducted in a 14-bed surgical pediatric cardiovascular intensive care unit (pCVICU) of a tertiary teaching hospital Necker Enfants Malades, Paris in France from July 2011 to December 2011. The Ethics committee of the Necker Enfants Malades Hospital approved the study provided that written and appropriate consent was obtained from the child’s parent(s) after they were informed of the objectives. We confirm that we have all necessary and appropriate consent from each child’s parents involved in the study, including consent to participate in the study and consent to publish.

All consecutive children aged less than 18 years, weighing more than 1,200 g, and requiring Ep infusion following CPB for open heart surgery were included. Non-inclusion criteria were unknown initial time infusion of Ep, unknown time of Ep flow-rate changes or unknown time of blood sampling. Children were enrolled prior to the onset of infusion and for a period lasting 6 hours after the start of Ep administration.

### Intervention

In the operating room, all children underwent endotracheal intubation and were mechanically ventilated under sedation, opioid treatment (midazolam and sufentanil) and neuromuscular blocking agent. Standard monitoring was used, comprising a radial or femoral artery catheter for measurement of systemic arterial blood pressure and intermittent blood sampling, a triple-lumen right internal jugular or femoral central venous catheter (CVC), and urinary bladder or rectal temperature probes. Normothermic CPB with intermittent warm blood cardioplegia was performed in every patient during the study period, except in cases where deep hypothermic circulatory arrest was indicated. Conventional ultrafiltration was performed during the CPB.

Ep infusion was initiated in the operating room (defined as time = 0 minutes), in association with milrinone at the end of the CPB, according to the local protocol and the risk of developing an LCOS: risk adjustment for congenital heart surgery 1 (RACHS-1) category, aortic cross-clamping duration, preoperative left ventricle dilatation, preoperative or intraoperative arterial pulmonary hypertension defined by intra cardiac right to left shunt or pulmonary arterial pressure over 2/3 mean systemic arterial pressure, hemodynamic instabilities (defined by a variation greater than 20% of HR and/or MAP) and physiological status [2,10]. Cases involving sepsis or preoperative myocardial dysfunction requiring inotropic support were excluded.

Ep (adrenalin 1 mg⋅mL^-1^, Renaudin^TM^ diluted to 100 μg⋅mL^-1^ or 50 μg⋅mL^-1^ in Glucose 5% Baxter^TM^, UK) was infused using a programmable electric syringe pump (DPS, Fresenius Vial^TM^, Brezins, France) through a triple-lumen central venous catheter (Arrow, Teleflex^TM^, PA 19605, USA) with a flow rate varying from 0.3 mL⋅h^-1^ to 1 mL⋅h^-1^. The latter was adjusted for age and hemodynamic objectives, namely: normal HR, normal MAP, normal time capillary refill, normal pulse, normal urine output (>2 mL⋅kg^-1^⋅h^-1^), ScVO2 >70% when measured, normal transthoracic echocardiography of left ventricular ejection fraction (60 to 80%) and normal plasma lactate level (<2.2 mmol⋅L^-1^) [2,11]. Depending on the congenital heart defects and the repair, the preload was normalized based on CVP and/or left atrial pressure (LAP). After normalized preload, intravenous milrinone at a dose ranging from 0.3 to 0.5 μg⋅kg^-1^⋅min^-1^ was systematically combined with Ep infusion without loading bolus at initiation.

Upon arrival to the ICU, medications were adjusted by the bedside nurse under the direction of the medical team: blood transfusion to reach a hemoglobin level above 10 g⋅dL^-1^, furosemide to maintain water balance and urine output over 2 mL⋅kg^-1^⋅h^-1^. Adequate analgesia and sedation were ensured by, respectively, continuous intravenous morphine or sufentanil and midazolam, mechanical ventilation with adequate pressure levels and oxygen inspired fraction and inhaled nitric oxide in case of pulmonary arterial hypertension.

The daily amount of intravenous glucose was adjusted for age: birth to 12 months: 4 g⋅kg^-1^⋅day^-1^, 12 months to 48 months: 3 g⋅kg^-1^⋅day^-1^, 48 months to 72 months: 2.5 g⋅kg^-1^⋅day^-1^ and over 72 months: 2 g⋅kg^-1^⋅day^-1^.

LCOS was defined if Ep and/or milrinone were needed over 48 hours to maintain normal hemodynamic parameters (normal HR, normal urine output, normal MAP, normal capillary refill time, warm extremities) without metabolic acidosis (standard plasma bicarbonate (HCO_3_^–^) level less than 22 mmol⋅L^-1^ or an increase in plasma lactate level greater than 2.2 mmol⋅L^-1^) [2,11]. In this study, no other catecholamines or corticosteroid was used in the first 6 hours following open heart surgery.

### Blood sampling

An initial blood sample (C_0_) was collected prior to CPB after which Ep infusion was initiated. A second blood sample (C_1_) was drawn at least 60 minutes after initiating Ep infusion. A last blood sample (C_2_) was drawn 40 minutes after a change in rate flow or prior to 6 hours after beginning Ep infusion in the case of a constant flow rate.

The 60-minute steady-state interval was chosen according to at least five times the Ep plasma half-life in healthy subjects (approximately 20 minutes) and the dead volume of the CVC used to infuse Ep at 0.3 to 1 mL⋅h^-1^ rate flow (approximately 40 minutes) [4].

C_0_ was used to assess plasma levels of endogenous Ep. Only C_0_ and C_1_ were drawn in patients who weighed less than 2,500 g, according to the percentage of blood volume permitted by the Ethics Committee of our institution.

### Sample handling

Blood assigned to catecholamine assays was sampled in EDTA-tubes placed in an ice bucket then immediately centrifuged at 3,000 g for 5 minutes. The plasma samples were then separated and immediately stored at -20°C and thereafter at -80°C before 24 hours running.

### Assay

Ep concentrations were blindly determined by means of HPLC with colorimetric detection [12]. After thawing, the volume of each sample was adjusted to 4 mL by adding distilled water and the internal standard, dihydroxybenzylamine. A 20-μL aliquot at 10°C was then injected into the chromatographic system comprised of a column (25 cm × 4.6 mm inner diameter, 5 μm Supelcosil LC-18 Supelco^TM^), an electrochemical ESA colorimetric detector (Model Coulochem III, Eurosep^TM^), dual analytic cells (ESA cell Model 5011) set at -0.05 V for the first detector and -0.3 V for the second detector, and a conditioning cell set at +0.3 V. The mobile phase, at 1.2 mL⋅min^-1^, consisted of a mixture of an aqueous acidic buffer containing heptane sulfonic acid and acetonitrile (93:7 v/v). Ep calibration curves were prepared according to the same procedure (2.5 μg to 75 μg/4 mL distilled water). The measured Ep concentration in pmol⋅mL^-1^ was converted to μg⋅L^-1^. The detection threshold (defined by variability <10% between measurements) for HPLC was 0.2 pmol⋅mL^-1^. Endogenous and exogenous Ep were strictly identical with regard to chromatographic detection.

### Patient data

Baseline patient characteristics were recorded, including non-cardiac medical history, gender, age, BW, RACHS-1 category, type of congenital heart defect, preoperative cyanotic status and left ventricular ejection fraction, duration of CBP and aortic cross-clamping, duration of pCVICU stay, mechanical endotracheal ventilation duration and death during pCVICU stay. Duration of both Ep and milrinone infusion were recorded. Variation of infused doses was recorded during the first 6 hours.

HR (beats⋅min^-1^) and invasive MAP (mmHg) data were recorded prior to CPB, at initiation of Ep, and then every 10 minutes for the first hour and thereafter every hour or less if needed during the subsequent 6 hours. Left ventricular ejection fraction (%) was measured at least once during the 6 hours. CVP (mmHg) was systematically recorded as well as LAP (mmHg) when measured. Temperature (°C) and urine outputs (mL⋅kg^-1^⋅h^-1^) were recorded during 6 hours following CBP.

Plasma lactate and glucose levels (mmol⋅L^-1^) were recorded before surgery and at least once thereafter during the following 6 hours. Arterial pH, ionized plasma calcium levels (mmol⋅L^-1^) and plasma HCO_3_^–^ levels (mmol⋅L^-1^) were recorded during the first 6 hours.

Results are expressed as raw numbers (%) or medians (ranges). The non-parametric Wilcoxon test was performed to compare pharmacokinetic and pharmacodynamic values before and under Ep infusion. *P* <0.05 was considered statistically significant.

### Pharmacokinetic-pharmacodynamic modeling

Ep concentration time-courses were described by a one-compartment open model with first-order elimination with the parameters of elimination clearance (CL) and volume of distribution (V). The differential equation connected to this model is thus,

(1) $$\mathrm{dA}\left(t\right)/\mathrm{dt}=\mathrm{Rate}\;–\;\mathrm{CL}.C\left(t\right)$$

(2) $$\mathrm{with}\;C\left(t\right)=A\left(t\right)/V$$

where A(t) and C(t) denote the amount of drug and concentration of drug in the body at time t. The endogenous production rate, q0, was taken into account in the model as follows,

(3) $$C\left(t\right)=A\left(t\right)/V+q0/\mathrm{CL}$$

The effect of BW was investigated in the pharmacokinetic model via an allometric relationship [13].

(4) $$P=P_{\mathrm{TYP}}{\left(\mathrm{BW}\right)}^{\mathrm{PWR}}$$

where P, P_TYP_ and PWR are the individual parameter, typical parameter and power exponent, respectively. The PWR exponent was estimated in a first attempt and then eventually fixed to ¾ for CL and q0 terms according to the typical weight-based allometric rule.

The circulating volume, V_Circ_ (L), was related to BW as follows [14].

(5) $$V_{\mathrm{Circ}}=0.08.\mathrm{BW}$$

As kinetics data were best described by a one-compartment model with first-order elimination, the half-life (T_½_) was related to V and CL as T_½_ = ln2 .V/CL = 0.69.V/CL.

The HR response, HR(t), was related to the Ep concentration via an *Emax* model.

(6) $$\mathrm{HR}\left(t\right)={\mathrm{HR}}_{0}+\left({\mathrm{HR}}_{\text{max}}-{\mathrm{HR}}_{0}\right).C\left(t\right)/\left\{C\left(t\right)+C_{50}\mathrm{HR}\right\}$$

where HR_max_ and HR_0_ are respectively the maximal and basal HR values and C_50_HR the concentration that induces 50% of the maximal effect on HR.

The MAP(t) is then expressed as:

(7) $$\mathrm{MAP}\left(t\right)=\mathrm{HR}\left(t\right).\mathrm{SV}.\mathrm{SVR}\left(t\right)+\mathrm{CVP}$$

where SV, SVR and CVP represent stroke volume, systemic vascular resistance and central venous pressure, respectively [15]. As SV and SVR were not known, the SV.SVR product variation was estimated via the function.

(8) $$\begin{array}{ll} \mathrm{SV}.\mathrm{SVR}\left(t\right)= & \mathrm{SV}.{\mathrm{SVR}}_{0}+\left(\mathrm{SV}.{\mathrm{SVR}}_{\text{max}}-\mathrm{SV}.{\mathrm{SVR}}_{0}\right).C\left(t\right)/\\ & \left\{C\left(t\right)+C_{50}\mathrm{SV}.\mathrm{SVR}\right\} \end{array}$$

where SV⋅SVR_0_, SV⋅SVR_max_ and C_50_SV⋅SVR respectively denote the SV⋅SVR product basal value, the product’s maximal value and the concentration that induces 50% of the maximal effect on SV⋅SVR.

Plasma glucose and lactate, G(t) and L(t), variations were modeled by a turnover model in which the stimulation of plasma glucose production, S(t), was related to Ep concentration as follows.

(9) $$\mathrm{dG}\left(t\right)/\mathrm{dt}=R_{\mathrm{GLY}}.S\left(t\right)–k_{\mathrm{GLY}}.G\left(t\right)\;$$

with

(10) $$S\left(t\right)=1+G_{\text{max}}C\left(t\right)/\left\{C\left(t\right)+C_{50}\mathrm{GLY}\right\}$$

where R_GLY_ and k_GLY_ represent the plasma glucose zero-order rate production and first-order elimination rate constant. G_max_ and C_50_GLY denote respectively the maximal stimulation effect and the Ep concentration that produces 50% of the maximal response.

The rate of change in plasma lactate level, dL(t)/dt, was related to the plasma glucose level variation rate as:

(11) $$\mathrm{dL}\left(t\right)/\mathrm{dt}=k_{\mathrm{GLY}}.G\left(t\right)–k_{\mathrm{LAC}}.L\left(t\right)$$

where k_LAC_ is the plasma lactate elimination rate constant.

Before Ep infusion, the systems are assumed to be at steady-state,

G(0) = GLY_0_, L(0) = LAC_0_, then k_GLY_ and k_LAC_ are

(12) $$k_{\mathrm{GLY}}=R_{\mathrm{GLY}}/{\mathrm{GLY}}_{0}$$

(13) $$k_{\mathrm{LAC}}=k_{\mathrm{GLY}}.{\mathrm{GLY}}_{0}/{\mathrm{LAC}}_{0}$$

where GLY_0_ and LAC_0_ denote respectively, basal plasma glucose and lactate levels.

### Population pharmacokinetic-pharmacodynamic analysis

Drug concentrations and responses were analyzed using a population approach, that is, a non-linear mixed-effect modeling approach. Data were analyzed using the MONOLIX software version 4.13 s [16] and the SAEM algorithm [17]. Differential equations were written in an MLXTRAN script file in MONOLIX to estimate the parameters. Residual variabilities were described by additive, proportional or exponential error models depending on the observation. An exponential model was used for between-subject variability (BSV). The effect of a covariate on a structural parameter was retained if it caused a decrease in the Bayesian information criterion (BIC) and/or reduced the corresponding BSV with *P* <0.05. Only covariates with a plausible effect on pharmacokinetic and pharmacodynamic parameters were investigated. The main covariates of interest in this pediatric population were BW and age.

### Visual predictive check (VPC) evaluation

Plasma Ep concentration, HR, MAP, plasma glucose and lactate-level time course was simulated from the respective final population model and compared with the observed data to evaluate the predictive performance of the model. The vector of pharmacokinetic parameters from 400 replicates of the database was simulated using the final model. Each vector parameter was drawn in a log-normal distribution with a variance corresponding to the previously estimated BSV. A simulated residual error was added to each simulated concentration. The 5th, 50th and 95th percentiles of the simulated dependent variables at each time point were then overlaid on the observed data and a visual inspection was performed. Because the patients received different Ep regimens, the Uppsala correction was used to produce the VPC plots [18].

### Evaluation and validation

Diagnostic graphics were used for evaluation of the goodness-of-fit. Concentration and effects profiles were simulated and compared with the observed data with the aid of the VPC in order to validate the model.

## Results

### Patients

A total of 55 children were initially enrolled, of which 16 patients were subsequently excluded: 6 because of incomplete parental consent, 7 because of missing C_1_ and C_2_ blood samples and 2 because of hemolysis. 

Hence, 39 children were included in the study. C_0_ samples were obtained in 33 patients, C_1_ in all children and C_2_ in 25 children for a total of 97 observations. Hemodynamic data (HR, MAP) and metabolic effects of Ep infusion (plasma lactate and glucose levels) were available in 38 children with 434, 464, 101 and 140 observations, respectively.

Five premature children with a gestational age <37 weeks (n = 1 at 33 weeks, n = 1 at 34 weeks, and n = 3 at 36 weeks) were recorded. Chromosomal disorders were reported in eight children (n = 3 with Down syndrome, n = 1 with di George syndrome, n = 1 with Loeys-Dietz syndrome, and n = 1 with Noonan syndrome n = 2 with suspected Noonan syndrome). Respiratory disorders were noted in seven patients (n = 3 with asthma, n = 3 with laryngotracheomalacia, and n = 1 with chronic aspiration pneumonia) and malnutrition (<2 SD) was observed in nineteen children.

Six children were treated before open heart surgery with converting enzyme inhibitors because of left ventricular dilatation, seven were treated with prostaglandins because of ductus arteriosus-dependent heart disease, and β-blockers were co-administered to three children because of obstruction of the left ventricular outflow track.

All children had transthoracic echocardiography prior to CPB; left ventricular ejection fraction was evaluated at 60% (25 to 78) with normal values (>50%) observed in 34 patients (87%). Ventricular diastolic function was not assessed. Eleven children were cyanotic (SpO2 <90%) prior to the surgery because of their congenital heart disease. Open heart surgeries were as follows: arterial switch (n = 9), repair of complete atrioventricular canal (n = 6), repair of ventricular septal defect (n = 6), total repair of tetralogy of Fallot (n = 4), pulmonary valvuloplasty (n = 3), repair of coarctation and ventricular septal defect closure (n = 2), repair of interrupted aortic arch with ventricular septal defect closure (n = 2), repair of double-outlet right ventricle (n = 2), repair of pulmonary artery stenosis (n = 2), pulmonary valvular replacement (n = 2), and repair of truncus arteriosus (n = 1). Surgery with deep hypothermic circulatory arrest was necessary in 11 children.

In the operating room all children required the following: a red blood cell transfusion, fresh-frozen plasma administration, neuromuscular blocking agents, and hypnotic and opioid drug infusion. Ultrafiltration of 650 mL (250 to 1,200) of fluid during CPB was performed to achieve a negative fluid balance and hematocrit at 44% (35 to 47). Milrinone and Ep were initiated just before weaning from CPB, with an infused Ep dose of 0.07 μg⋅kg^-1^⋅min^-1^ (0.01 to 0.23) and an infusion milrinone dose of 0.5 μg⋅kg^-1^⋅min^-1^ (0.2 to 0.7). Milrinone infusion was stopped after 1.5 days (1 to 13).

Delayed sternal closure occurred in four patients. Postoperative left ventricular ejection fraction under Ep and milrinone infusion was at 60% (30 to 70) with normal values (>50%) observed in 34 patients (87%). CVP and LAP were 11 mmHg (8 to 15) and 8 mmHg (6 to 14), respectively. Four children exhibited supraventricular tachycardia, one had ventricular tachycardia and five had a transient atrioventricular block, which required external cardiac pacing. Urine output was 4.5 mL⋅kg ⋅h^-1^ (0.8 to 7.5) and pH, plasma HCO_3_^–^ (mmol⋅L^-1^) and plasma ionized calcium (mmol⋅L^-1^) levels were: 7.39 (7.27 to 7.45); 24 (20 to 26) and 1.28 (1.12 to 1.5), respectively. All children required diuretics, whereas none were under corticosteroid therapy during the 6 hours following surgery. Ten children needed inhaled nitric oxide for pulmonary arterial hypertension during the postoperative course. Endotracheal mechanical ventilation was performed for all patients during 2.1 days (1 to 17). None of the patients required renal replacement therapy and none had liver injury according to prothrombin activity and/or Factor V levels (lower than 50% for at least 24 hours). None of the children died during their pCVICU stay. Finally, nine children (23%) had LCOS according to the classical definition. Table 1 summarizes overall patient characteristics.

**Table 1:** Patient characteristics and baseline kinetic and dynamic data

**Patient characteristics (n = 39)**	**Values**
**Demographics**	
Age, months	3.9 (0.1 to 189)
Gender, male, n (%)	26 (66.6%)
Body weight, kg	4.5 (2.5 to 58)
**Preoperative physiological profile**	
RACHS-1, categories, median (range)	3 (2 to 4)
Category 2, n	16
Category 3, n	17
Category 4, n	6
Prothrombin activity (%)	80 (50 to 100)
Creatinine clearance, mL⋅min^-1^⋅1.73 m^(2) -1^	91 (22 to 200)
**Per-operative course**	
Duration of CPB, minutes	107 (52 to 222)
Aortic cross-clamping time, minutes	64 (9 to 140)
**Postoperative course**	
Ep infusion duration, days	1.5 (1 to 13)
pCVICU length of stay, days	3 (2 to 23)
**Baseline kinetic and dynamic parameters**	
Ep concentration, μg⋅L^-1^	0.062 (0.037 to 0.25)
Heart rate, beats⋅min^-1^	135 (70 to 180)
Mean arterial pressure, mmHg	51 (25 to 65)
Plasma glucose level, mmol⋅L^-1^	6.2 (4 to 10.1)
Plasma lactate level, mmol⋅L^-1^	1 (0.5 to 3)

RACHS, risk adjustment for congenital heart surgery; n, number; CPB, cardiopulmonary bypass; min, minutes; Ep, epinephrine; pCVICU, pediatric cardiovascular intensive care unit. Values represent median and the presentation of the value in parentheses defined the range from (min to max).

### Epinephrine pharmacokinetics

The increase in Ep concentration during infusion was significant: 2.94 μg⋅L^-1^ (0.37 to 71) compared to baseline Ep concentration, 0.062 μg⋅L^-1^ (0.037 to 0.25) (*P* <0.001). A one-compartment open model with linear elimination adequately described the Ep time courses. An additional movie file shows this in more detail (see Additional file 1). The pharmacokinetic parameters were V, CL and q0. The residual variability was ascribed to a proportional model. BW was the main covariate influencing CL and q0 (*P* <0.001). Both PWR estimates for q0 and CL were close to 1 (0.98 and 0.985), however CL and q0 were poorly estimated (relative standard errors near 50%). Moreover, BIC decreased from 214.2 to 205.0 when PWR was estimated and further decreased to 199.0 when PWR was fixed to ¾. Also, there was no visible or significant difference between the two models on the observed versus predicted plots. An additional movie file shows this in more detail (see Additional files 2 and 3).

Hence, these power values were fixed to ¾ according to the BW-based allometric rule. V was assumed to be equal to the circulating volume because V could not be accurately estimated and because of the hydrophilic nature of Ep. No other covariate (gender, pH, temperature, RACHS-1 categories, creatinine clearance, gestational age, cyanosis, malnutrition) influenced the pharmacokinetics. The final relationship for Ep CL and q0 was:

CL(BWi) = θ_CL_ × (BWi)^3/4^ and q0(BWi) = θ_q0_ × (BWi )^3/4^, then

θ_CL_ (L⋅h^-1^⋅kg^-1^) = 2, θ_q0_ (μg⋅h^-1^⋅kg^-1^) = 0.15

where θ_CL_ and θ_q0_ are typical unit clearance and endogenous production rate. For an individual weighing 10 kg, Ep CL, q0, V and T½ were:

CL (10 kg) = 2 × 10^¾^ = 11.2 L⋅h^-1^, q0 (10 kg) = 0.15 × 10^¾^ = 0.84 μg⋅h^-1^, V(10 kg) = 0.08 × 10 = 0.8 L and T_½_(10 kg) = (ln2 × V(10 kg) /CL(10 kg)) × 60 = 3 minutes.

**Figure 1:**
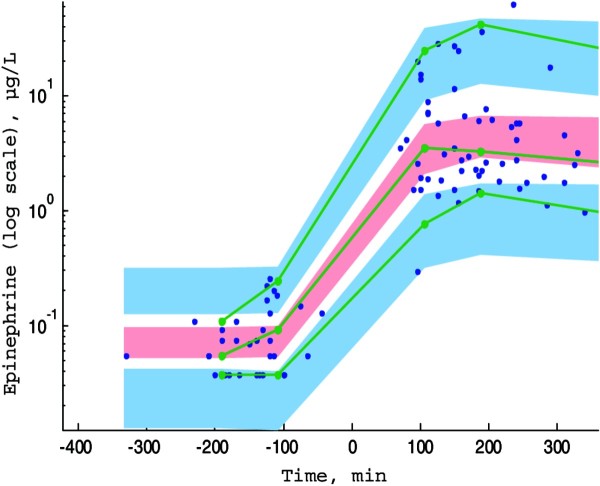
**Prediction corrected-visual predictive check (PC-VPC) for epinephrine concentrations versus time in minutes.** The green lines depict the 5th, 50th and 95th percentiles of observed data; the areas represent the 95% confidence interval around the simulated percentiles. Blue color represent the 5th and the 95th percentile of the predicted concentration versus time and pink color represent the median predicted concentration versus time. Time 0 min represents the starting time of epinephrine infusion.

Table 2 summarizes the final population estimates. All parameters were estimated with good precision. Figure 1 depicts the VPC and shows that the observed concentrations were well centered around the simulated median predictions.

**Table 2:** Population pharmacokinetic parameters

**Parameters**	**Estimate**	**RSE (%)**
θ_CL_ (L⋅h^-1^⋅kg^-1^)	2	17
θ_BW_ (CL(BW_i_) = θ_CL_ × BW_i_^3/4^ )	0.75 (fixed)	NA
θ_q0_ (μg⋅h^-1^⋅kg^-1^)	0.15	19
θ_BW_ (q0(BW_i_) = θ_q0_ × BW_i_^3/4^ )	0.75 (fixed)	NA
V (L) for a 10 Kg individual	0.8	NA
T_½_ (min), for a 10 Kg individual	3	NA
η_CL_ (square root of ω^2^_CL_)	1	14
η_q0_ (square root of ω^2^_q0_)	1.1	13
Residual variability (proportional)	0.3	15
Correlation (η_CL_, η_q0_)	0.88	5

The volume of distribution of epinephrine was ascribed to the circulating volume, estimated as a function of bodyweight (see Materials and methods). CL, elimination clearance; q0, endogenous production rate; V, volume of distribution; CV, circulating volume; θ_CL_, typical unit clearance; θ_q0_,typical unit endogenous production rate; θ_BW_, bodyweight influential parameter; T_½_, half-life.

RSE, relative standard error; η, between-subject variability (BSV); BW, bodyweight; NA, not applicable.

### Epinephrine hemodynamics

**Figure 2:**
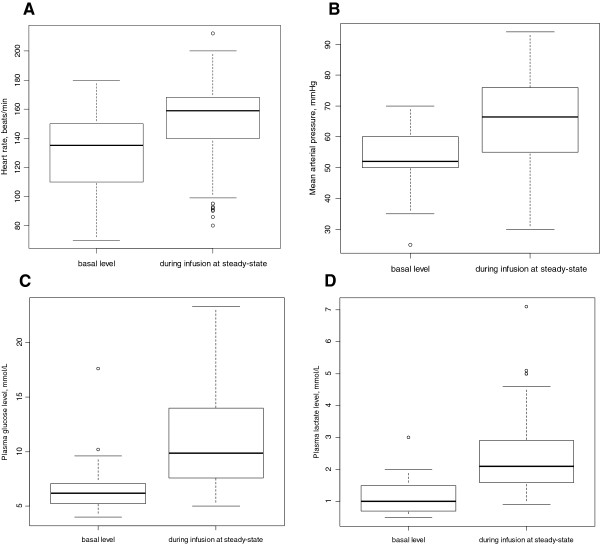
**Box and whisker plots of hemodynamic and metabolic data before and during epinephrine infusion.** Box and whisker plots of (i) heart rate, beats⋅min^-1^**(A)** before epinephrine infusion (minimum = 70; maximum (max) = 180; median = 135) and during epinephrine infusion (minimum = 70; max = 212; median = 159) (variance (w) = 2,563; *P* = 2.10^-8^); (ii) mean arterial pressure, mmHg **(B)** before epinephrine infusion (minimum = 25; max = 65; median = 51) and during epinephrine infusion (minimum = 30; max = 94; median = 66) (variance (w) =2613; *P* = 5.10^-11^); (iii) plasma glucose, mmol⋅L^-1^**(C)** before epinephrine infusion (minimum = 4; max = 10.1; median = 6.2) and during epinephrine infusion (minimum = 4.9; max = 23.3; median = 9.85) (variance (w) = 339; *P* = 6.10^-9^) and (iv) lactate levels, mmol⋅L^-1^**(D)** before epinephrine infusion (minimum = 0.5; max = 3; median = 1) and during epinephrine infusion (minimum = 0.9; max = 7.1; median = 2.1) (variance (w) = 218; *P* = 3.10^-10^).

After initiation of Ep infusion, (i) HR increased significantly from 135 beats⋅min^-1^ (70 to 180) to 159 beats⋅min^-1^ (80 to 212) (variance (w) = 2563; *P* = 2.10^-8^), (ii) MAP values increased significantly from 51 mmHg (25 to 65) to 66 mmHg (30 to 94) (variance (w) = 2613; *P* = 5.10^-11^) (Figure 2). The variations in HR and MAP as a function of Ep concentration were well explained by the Emax models, expressed by equations (6) to (8). The residual variability was ascribed to a proportional model. BSVs were estimated for HR_0_, C_50_HR, SV.SVR_0_ and SV.SVR_max_. Age was the main covariate influencing HR_0_ and SV⋅SVR_0_ (*P* <0.001) where HR_0i_ = HR_0_ × age_i_^-0.0612^ and SV.SVR_0i_ = SV⋅SVR_0_ × age_i_^0.094^ respectively. Including age in the model dramatically decreased the BIC (from 5,998 to 5,971) and improved the curve-fitting of the model. In addition, RACHS-1 category was significant (*P* = 0.04) in the estimation of SV⋅SVR_max_: 0.44 and 0.26 for RACHS-1 categories 2 and 3 to 4 respectively; the BSVs for HR_0,_ C_50_HR, SV⋅SVR_0_ and SV⋅SVR_max_ varied from 0.19, 1.0, 0.25 and 0.32 (basic model) to 0.14, 1.22, 0.13 and 0.23 (final model). Also, the BIC decreased from 5,971 (including age) to 5,965 (final model).

**Figure 3:**
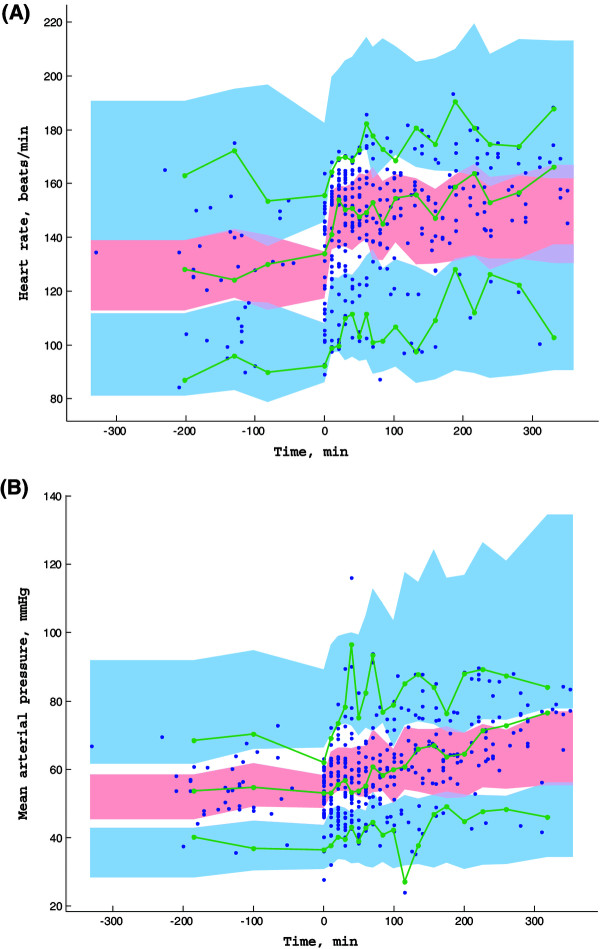
**Prediction corrected-visual predictive check (PC-VPC) for heart rate (A) and mean arterial pressure (B) observations versus time in minutes.** The green lines depict the 5th, 50th and 95th percentiles of observed data; the areas represent the 95% confidence interval around the simulated percentiles. Blue color represent the 5th and the 95th percentile of the predicted HR and MAP versus time and pink color represent the median predicted HR and MAP versus time. Time 0 min represents the starting time of epinephrine infusion.

No other covariate improved the model (including duration of aortic cross-clamping, duration of CPB, deep hypothermia and milrinone dosage). The final population parameters are summarized in Table 3. The VPC plots in Figure 3 show that the observed HR and MAP values are well-centered around the predicted median of the model.

**Table 3:** Hemodynamic and metabolic population parameters

**Hemodynamic population parameters**	**Estimate**	**RSE (%)**
HR_0_ (b⋅min^-1^)	133	3
θ_age_ (HR_0i_ = HR_0_ x age_i_^-0.061^)	-0.061	18
HR_max_ (b⋅min^-1^)	180	3
C_50_ HR (μg⋅L^-1^)	5.71	37
SV⋅SVR_0_	0.31	3
θ_age_ (SV⋅SVR_0i_ = SV⋅SVR_0_ x age_i_^0.094^ )	0.094	14
SV⋅SVR_max_, RACHS-1 = category 2	0.44	20
SV.SVR_max_, RACHS-1 = categories 3,4	0.26	20
C_50_ SV⋅SVR (μg.L^-1^)	18	59
η_HR0_ (square root of ω^2^_HR 0_)	0.14	14
η_C50 HR_ (square root of ω^2^_C 50 HR_)	1.22	17
η_SV.SVR0_ (square root of ω^2^_SV.SVR0_)	0.13	17
η_SV.SVRmax_ (square root of ω^2^_SV.SVRmax_)	0.23	42
Residual variability (proportional)		
HR	0.08	4
MAP	0.16	4
**Metabolic population parameters**		
GLY_0_ (mmol⋅L^-1^)	5.46	5
G_max_	1.69	6
R_GLY_ (mmol⋅L^-1^.min^-1^)	0.04	25
C_50_GLY (μg⋅L^-1^)	0.52	9
LAC_0_ (mmol⋅L^-1^)	1.23	7
η_GLY0_ (square root of ω^2^_GLY 0_)	0.21	23
η_Gmax_ (square root of ω^2^_Gmax_)	0.213	26
η_RGly_ (square root of ω^2^_RGLY_)	1	21
η_LAC0_ (square root of ω^2^_LAC0_)	0.33	18
Residual variability (constant additive)		
GLY (mmol⋅L^-1^)	2.23	5
LAC (mmol⋅L^-1^)	0.5	11

HR_0_, basal heart rate; HR_max_, maximal HR; C_50_HR, Ep concentration producing 50% of HR _max_; SV⋅SVR, product of stoke volume by systemic vascular resistances; SV⋅SVR_0_, basal SV⋅SVR value; SV.SVR_max_, maximal SV⋅SVR value; C_50_SV⋅SVR_,_ Ep concentration producing 50% of SV⋅SVR_max_; RACHS-1, risk adjustment for congenital heart surgery 1; GLY_0_, basal plasma glucose level; R_GLY_, glucose zero-order production rate; G_max_, relative maximal increase in R_GLY_; C_50_GLY, Ep concentration that produces 50% of the maximal response on plasma glucose level; LAC_0_, basal plasma lactate level; RSE, relative standard error; η, between-subject variability (BSV); θ_age_, age influential parameter.

### Metabolic effects of epinephrine

**Figure 4:**
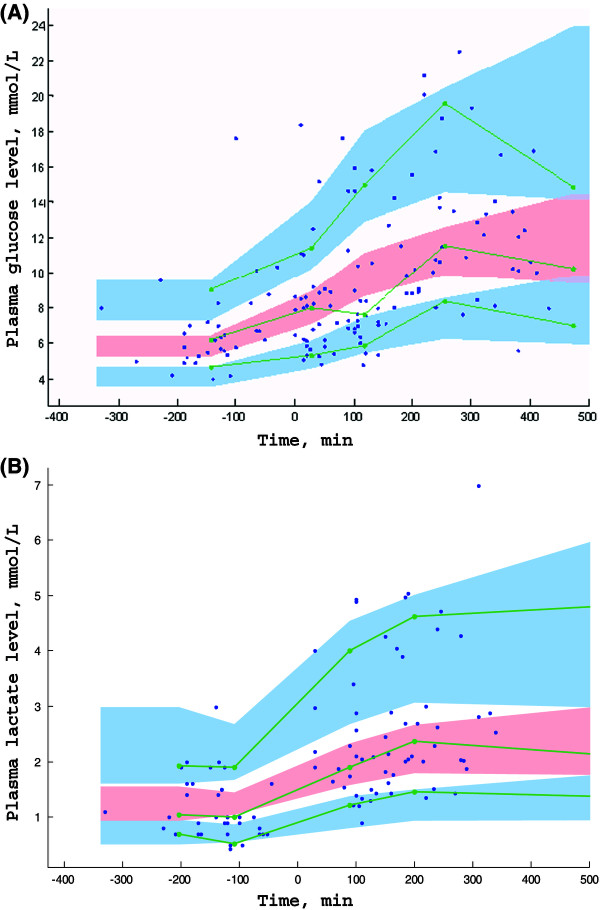
**Prediction corrected-visual predictive check (PC-VPC) for plasma glucose level (A) and plasma lactate level (B) observations versus time in minutes.** The green lines depict the 5th, 50th and 95th percentiles of observed data; the areas represent the 95% confidence interval around the simulated percentiles. Blue color represent the 5th and the 95th percentile of the predicted plasma glucose level and lactate level versus time and pink color represent the median predicted plasma glucose level and lactate level versus time. Time 0 min represents the starting time of epinephrine infusion.

Both plasma glucose and lactate levels increased significantly after the initiation of Ep infusion from 6.2 mmol⋅L^-1^ (4.0 to 10.1) and 1 mmol.L^-1^(0.5 to 3) to 9.85 mmol.L^-1^ (4.9 to 23.3) [variance (w) = 339; *P* = 6.10^-9^] and 2.1 mmol⋅L^-1^(0.9 to 7.1) (variance (w) = 218; *P* = 3.10^-10^), respectively (Figure 2). Assuming that Ep stimulated the glucose production rate, the turnover models expressed by equations (9) to (13) effectively described the variations in plasma glucose (G(t)) and lactate levels (L(t)). There was no significant covariate effect, including those of exogenous glucose supply, age or BW. The residual variability was ascribed to a constant additive model. BSVs were estimated for GLY_0_, R_GLY_, G_max_ and LAC_0_. All parameters were well-estimated with low relative standard errors. Table 3 summarizes the population estimates. The VPC plots in Figure 4 show that the observed plasma glucose and lactate levels are well-centered around the predicted median of the model.

### Epinephrine dosing simulations

**Figure 5:**
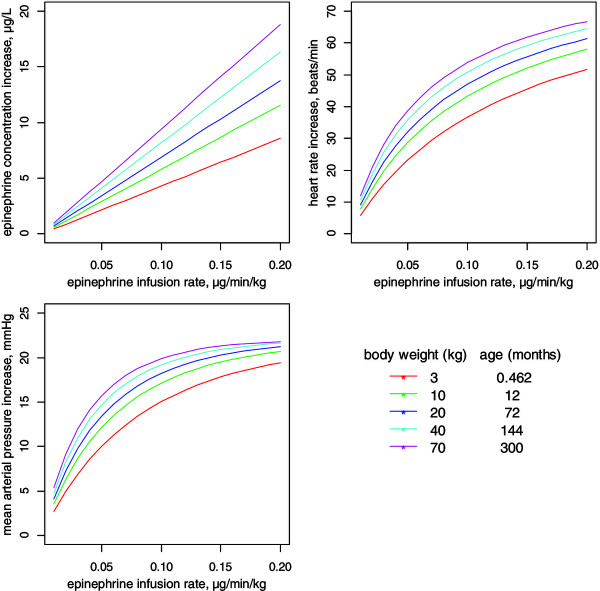
Dosing simulations depicting the increases in epinephrine concentration and hemodynamic responses as a function of administered infusion rate in children of different bodyweights and ages for patients with a risk adjustment for congenital heart surgery (RACHS)-1 category of 2.

**Figure 6:**
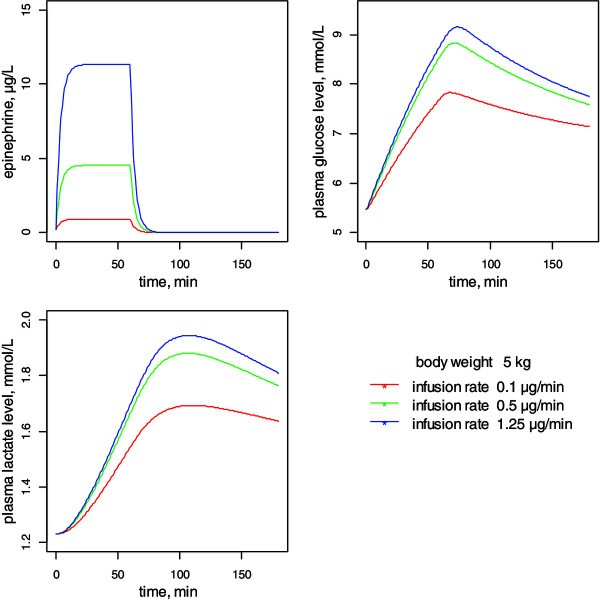
**Time-course simulations of epinephrine concentration, plasma glucose and lactate levels following 0.02 to 0.25 μg⋅min**^**-1**^**⋅kg**^**-1 **^**epinephrine infusions in a child weighing 5 kg.**

Using the hemodynamic model, the effects of various infusion rates of Ep on HR and MAP were assessed as a function of age and BW. As shown in Figure 5, the increase in Ep concentration versus infusion rate was linear although the increases in HR and MAP were curvilinear, due to the Ep concentration-Emax model. Similarly, Figure 6 shows the metabolic responses for a child weighing 5 kg with three infusion rates: 0.02, 0.1 and 0.25 μg⋅kg⋅min^-1^.

## Discussion

Little is known about the pharmacokinetics and pharmacodynamics of Ep in children although this drug is used in the pediatric population. The present study using a population approach adequately described the kinetics, hemodynamic and metabolic effects of Ep in critically ill children, highlighting the between-subject variabilities which were well explained by age and BW.

### Epinephrine pharmacokinetics

A one-compartment open model with linear elimination adequately described the data as previously reported [3,5,19]. The effect of BW using the allometric scale on clearance and Ep endogenous production improved the model and partly explained the between-subject variability [13]. This was not unexpected since endogenous rates of production and clearance of Ep are dependent on enzymatic maturation, both of which are related to age and BW [20]. With regard to endogenous Ep rate production, given that the concentrations observed following the infusion were well above 10-fold the baseline concentrations (approximately 50-fold), the contribution of possible variations in endogenous production was assumed to be negligible during the infusion. We could not adequately estimate volume of distribution because Ep concentration was measured only in the steady state; however, adjusting the volume of distribution to the circulating volume is justified considering the hydrophilic nature of Ep. We did not find any effect of creatinine clearance, as only 10% of Ep is excreted unchanged via the renal route and is mainly and rapidly inactivated by either methylation via the effect of catechol-*O*-methyl transferase or oxidative deamination by monoamine oxidase into inactive metabolites excreted by the kidney [21]. In contrast to the study of Abboud *et al*., neither RACHS-1 categories nor duration of CBP or aortic cross-clamping, which reflect the severity of illness, were found to be significant, possibly because of the small sample size and the difference in patient age and illness groups between the studies [6].

### Epinephrine - hemodynamic effects

To the best of our knowledge, this is the first study in which the hemodynamic responses to Ep in preventing LCOS were modeled. Only HR and MAP were recorded in this study. In adult volunteers, as in critically ill patients, Ep increases HR as well as MAP [8,22,23]. The predominant effect of Ep when administered at low dose is mediated by β-adrenergic receptors, which increase HR and SV [23-25]. The resulting hemodynamic response may differ in children because of (i) the relative immaturity of the myocardium, which precludes a significant increase in SV [26] and (ii) a variation in β1 and β2-adrenergic receptor density related to age [27]. Furthermore, newborn and infant cardiac outputs are more dependent on an increase in HR [25]. This is confirmed by our hemodynamic model, which highlighted the primary role of HR, which in turn influences MAP, that is, the C_50_ estimate for the SV⋅SVR product was three times as high as that estimated for HR, reflecting a much greater sensibility of the HR response to Ep.

The Emax models were effective in relating both HR and MAP responses to Ep concentration [28]. Measuring cardiac output is hazardous in children undergoing repair of congenital heart disease because of residual ventricular and/or auricular shunt. Hence, we could only estimate the SV⋅SVR product that relates MAP to HR. Moreover, as expected, age was found to be a significant covariate that dramatically improved the model: HR was negatively related to age whereas the SV⋅SVR product increased with age. This latter finding is easy to explain because both SV and MAP increase with age [29]. Likewise, RACHS-1 categories 3 and 4 decreased the maximal MAP response (SV⋅SVR_max_ product). This effect may be related to the role of systemic inflammatory syndrome following CB, which alters myocardial and vascular response to Ep [11]. The impact of temperature and pH on HR was also investigated but was not found significant.

As milrinone was infused in all children, this could have had a confounding effect on hemodynamic responses. There are conflicting data in the literature on the effect of milrinone on HR, namely an increased or unchanged HR [30]. In the present study, none of the parameters of the hemodynamic model were found to be influenced by a dose-dependent effect of milrinone. Moreover, any possible confounding effect would be negligible since an immediate increase in HR was observed after Ep initiation which is not compatible with the delayed response to milrinone [31].

### Epinephrine - metabolic effects

The turnover model herein provided a valid relationship between Ep concentration and plasma glucose and lactate levels, as these metabolic responses are dependent on stimulation of glycogenolysis via the activation of β_2_-adrenergic receptors [32]. During Ep infusion, the increases in plasma glucose and lactate levels were significant, albeit delayed as compared to the hemodynamic responses. Indeed, exogenous Ep has previously been shown to increase plasma glucose and lactate levels [8,33]. Lactate is mainly produced via the anaerobic glycolytic breakdown of glucose to pyruvate [32]. An excessive vasoconstriction mediated by α-adrenergic receptors results in lactate accumulation due to limited oxygen supply [34]. However, lactate may also accumulate during accelerated aerobic glycolysis driven by Ep [35], and it is unlikely that the rise in plasma lactate level is due to an excessive vasoconstrictor effect via Ep α-adrenergic receptor stimulation, given the low doses administered. Lastly, we believe that these increases are strongly related to Ep without confounding factors because (i) there was no significant effect of the exogenous supply of glucose, (ii) there were no other potential hyperglycemic treatments, such as corticosteroids and (iii) milrinone does not elevate glucose and/or lactate levels [36].

### Epinephrine dosing simulations

Using the final model, it was possible to highlight the differences in responses to a same infusion rate according to age or BW. Therefore, these simulations allow the determination of an a priori dosing schedule, for specific BW and age, to produce a suitable increase in HR and MAP. Interestingly, these plots clearly show that the amplitude of HR or MAP increase following various Ep infusion rates is related to the child’s BW, that is, the lower the BW, the smaller the amplitude of increase.

### Limitations of the study

The small sample size likely limited the identification of other significant covariates that could affect either the pharmacokinetics or the responses to Ep. We were not able to evidence a hemodynamic effect of milrinone in the model; however, we cannot ignore the potential effect of the latter and must assume that the modeling of the hemodynamic effects of Ep, including its simulations, implicitly take into account the effects of milrinone. Furthermore, such simulations need to be confirmed in a future clinical study. Finally, as only children who underwent an open heart surgery with CPB were included, our results cannot be easily extended to patients with other circulatory failure etiologies.

## Conclusion

This original study on pharmacokinetics, hemodynamic and metabolic effects of Ep to prevent postoperative LCOS in children highlights as expected, clear between-subject variability related to the substantial role of age and BW. Taking into account these individual characteristics should help clinicians in determining an appropriate a priori dosing regimen.

## Key messages

•In critically ill cardiac postoperative children, lower bodyweight was associated with lower epinephrine clearance.

•Differences of hemodynamic responses to epinephrine were related to age and bodyweight: the lower the bodyweight, the smaller the amplitude of heart rate and mean arterial pressure increase.

•Increase of plasma glucose and lactate levels was related to epinephrine concentration without any effect of age, bodyweight or exogenous glucose supply.

•Epinephrine dosing simulations should help the clinician in determining an appropriate a priori dosing regimen.

## Abbreviations

A: amount; BIC: Bayesian information criterion; BSV: between-subject variability; BW: bodyweight; C: concentration; C50GLY: epinephrine concentration that produces 50% of the maximal response on plasma glucose level; C50HR: epinephrine concentration that induces 50% of the maximal effect on heart rate; C50SV⋅SVR: epinephrine concentration that induces 50% of the maximal effect on stroke volume-systemic vascular resistance; Circ: circulating volume; CL: clearance; CPB: cardiopulmonary bypass; CVC: central venous catheter; CVP: central venous pressure; Ep: epinephrine; GLY: plasma glucose level; Gmax: maximal increase on glucose zero-order rate production; HPLC: high-performance liquid chromatography; HR: heart rate; HR0: basal heart rate; HRmax: maximal heart rate value; kGLY: first-order elimination rates; kLAC: plasma lactate elimination rate; LAC: plasma lactate level; LCOS: low cardiac output syndrome; MAP: mean arterial pressure; pCVICU: pediatric cardiovascular intensive care unit; PC-VPC: prediction corrected-visual predict check; PWR: power exponent; q0: endogenous production rate of epinephrine; RACHS-1: risk adjustment for congenital heart surgery 1; RGLY: plasma glucose zero-order rate production; SD: standard deviation; SV: stroke volume; SV⋅SVR0: basal value of stroke volume-systemic vascular resistance product; SV⋅SVRmax: maximal value of stroke volume-systemic vascular resistance product; SVR: systemic vascular resistance; t: time; T½: half-life; V: volume of distribution; θCL: typical unit clearance; θq0: typical unit endogenous production rate.

## Competing interests

The authors declare that they have no competing interests.

## Authors' contributions

MO collected and analyzed the data, and drafted the manuscript. SU analyzed the data and drafted the manuscript. OSV made substantial contributions to the analysis and interpretation of the data. AB and ID collected the data and were involved in revising the manuscript and PP was involved in critically revising the manuscript for important intellectual content. JMT conceived the study, participated in its design and coordinated and drafted the manuscript. All authors read and approved the final manuscript.

## Supplementary Material

Additional file 1 **Example of epinephrine concentration time courses in 16 individual fits.** We can see the satisfactory adequacy between predicted (green curve) and observed (blue cross) epinephrine concentration (μg⋅L^-1^) time (min) courses. Click here for file

Additional file 2 Population pharmacokinetics of epinephrine: estimation of the power coefficients for the bodyweight effect. Click here for file

Additional file 3 **Predicted versus observed plots for the fixed and estimated allometric power coefficients.** We can observe that there is no visible or significant difference between the two models. Click here for file
