# Supplementary figures and images for: Pharmacokinetics, hemodynamic and metabolic effects of epinephrine to prevent post-operative low cardiac output syndrome in children
Source: Crit Care. 2014 Jan 24;18(1):R23. doi: 10.1186/cc13707 (PMC4056810; doi:10.1186/cc13707)

**Example of epinephrine concentration (µg.L-1) time (min) courses in 16 individual fits
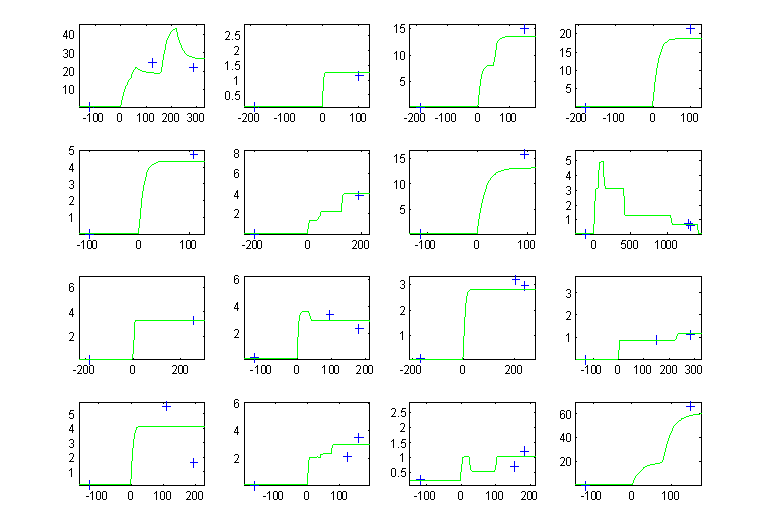
**

Supplement: Additional file 1 — Example of epinephrine concentration time courses in 16 individual fits. We can see the satisfactory adequacy between predicted (green curve) and observed (blue cross) epinephrine concentration (μg⋅L-1) time (min) courses. [file cc13707-S1.doc]
