# Supplementary material for: Pharmacokinetics, hemodynamic and metabolic effects of epinephrine to prevent post-operative low cardiac output syndrome in children
Source: Crit Care. 2014 Jan 24;18(1):R23. doi: 10.1186/cc13707 (PMC4056810; doi:10.1186/cc13707)
Supplement: Additional file 2 — Population pharmacokinetics of epinephrine: estimation of the power coefficients for the bodyweight effect. [file cc13707-S2.doc]

(i) Population pharmacokinetics of epinephrine: estimation of the power coefficients for the bodyweight effect.

| **Pharmacokinetic parameters** | **Estimate** | **RSE (%)** |
| --- | --- | --- |
| θ_CL_ (L.h^-1^.kg^-1^) | 1.31 | 42 |
| θ_BW_ (CL(BW_i_) = θ_CL_ x BW_i_^PWR^ ) | 0.98 | 22 |
| θ_q0_ (µg.h^-1^.kg^-1^) | 0.1 | 48 |
| θ_BW_ (q0(BW_i_) = θ_q0_ x BW_i_^PWR^ ) | 0.985 | 25 |
| V (L) for a 10 Kg individual | 0.8 | NA |
| T_½_ (min), for a 10 Kg individual | 2.4 | NA |
| η_CL_ (square root of ω^2^_CL_) | 1 | 12 |
| η_q0_ (square root of ω^2^_q0_) | 1.1 | 14 |
| Residual variability (proportional) | 0.3 | 15 |
| Correlation (η_CL_ , η_q0_) | 0.88 | 5 |

The volume of distribution of epinephrine was ascribed to the circulating volume, estimated as a function of bodyweight (see methods)

CL, elimination clearance; q0, endogenous production rate; V, volume of distribution; CV, circulating volume; θ_CL_, typical unit clearance; θ_q0_,typical unit endogenous production rate; θ_BW_, bodyweight influential parameter ; T_½_, half-life

RSE (%), relative standard error; η, between subject variability (BSV); BW, bodyweight; NA, not applicable
