# Supplementary material for: Pharmacokinetics, hemodynamic and metabolic effects of epinephrine to prevent post-operative low cardiac output syndrome in children
Source: Crit Care. 2014 Jan 24;18(1):R23. doi: 10.1186/cc13707 (PMC4056810; doi:10.1186/cc13707)
Supplement: Additional file 3 — Predicted versus observed plots for the fixed and estimated allometric power coefficients. We can observe that there is no visible or significant difference between the two models. [file cc13707-S3.doc]

(ii) Below: PRED (popPred) vs. OBS (DV) plots for the fixed (left) and estimated (right) allometric power coefficients.

.


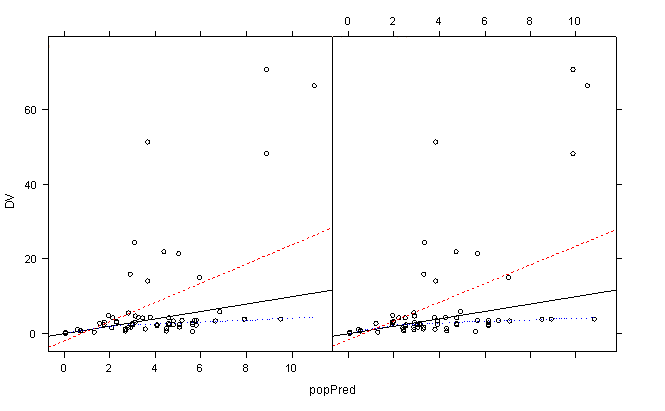


Key: black line, identity line; red dashed line, actual regression line; blue dashed line, spline
